# Adrenal cortical adenoma arising in the setting of renal–adrenal fusion: a case report and review of the literature

**DOI:** 10.1186/s13256-023-04287-0

**Published:** 2023-12-28

**Authors:** Samuel R. Wang, Richard Morris, Suad Taraif, Amandeep Aneja

**Affiliations:** 1 School of Arts and Sciences, Rutgers University, New Brunswick, NJ 08854 USA; 2 Office of The Chief Medical Examiner of The State of Maryland, Baltimore, MD 21223 USA; 3 Department of Pathology, Cooper University Health Care, Camden, NJ 08103 USA; 4 Department of Pathology, Cooper Medical School of Rowan University and Cooper University Health Care, Camden, NJ 08103 USA

**Keywords:** Renal–adrenal, Fusion, Adenoma

## Abstract

**Background:**

Renal–adrenal fusion is a rare entity resulting from incomplete encapsulation of the adrenal gland and kidney. Only 18 cases have been reported in English literature to date.

**Case presentation:**

Our patient is a 77-year-old African American female who presented with a systolic blood pressure of 200 mmHg. Computed tomography showed an enhancing 9 × 6 cm mass anterior and medial to the left kidney. The patient underwent a left adrenalectomy with partial nephrectomy. Gross and histologic examinations revealed an adrenal cortical adenoma and renal–adrenal fusion.

**Conclusion:**

Renal–adrenal fusion may pose a diagnostic challenge, particularly when there is a concurrent adrenal adenoma. It is important to be aware of this uncommon anomaly to avoid misdiagnosis and overtreatment.

## Background

Renal–adrenal fusion is a rare entity originally described by Rokitansky in 1855 [1]. It has been hypothesized that this anomaly is caused by failure of the retroperitoneal mesenchyme to stimulate capsule formation, thus impeding the encapsulation of the adrenal gland and kidney [2]. Renal–adrenal fusion causes no physiological symptoms, and a majority of the reported cases were discovered incidentally in nephrectomy specimens. However, renal–adrenal fusion may pose a diagnostic challenge, especially on preoperative imaging, and mischaracterization of this anomaly as a renal or adrenal malignancy can result in overtreatment.

## Case presentation

**Fig. 1:**
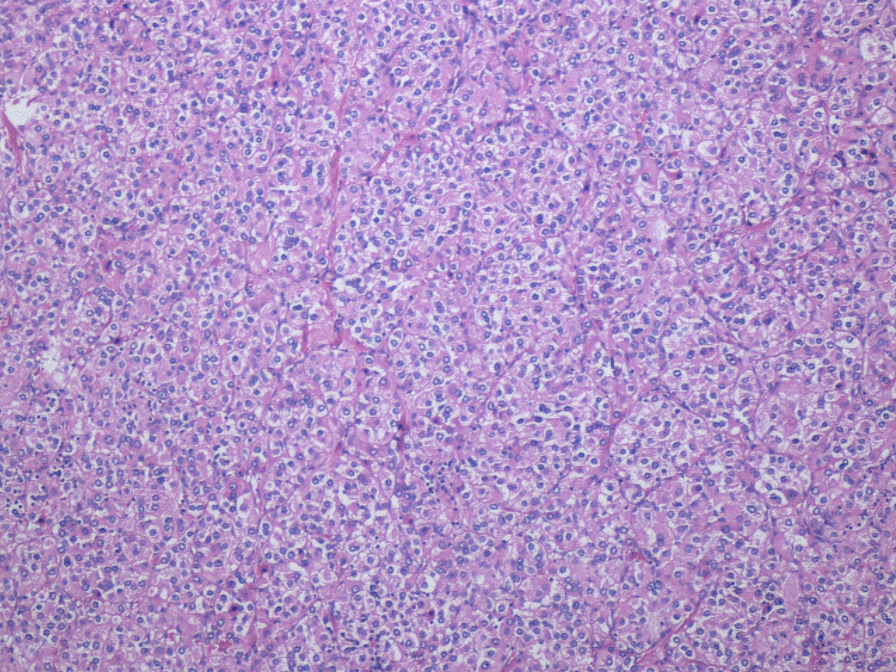
Histological findings of an adrenal cortical adenoma (hematoxylin and eosin staining, original magnitude ×100)

**Fig. 2:**
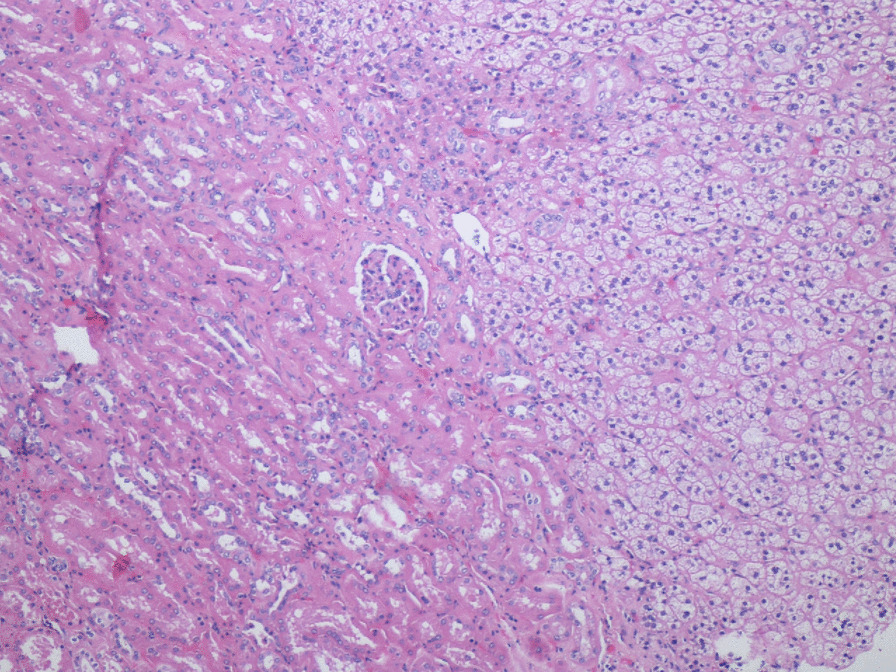
Histological findings of adherence of adrenal tissue to the renal cortex without intervening capsule (hematoxylin and eosin staining, original magnitude ×100)

A 77-year-old African American woman with a medical history of hypertension presented with a systolic blood pressure of 200 mmHg. There was no significant family or social history. A renal ultrasound showed a 12 × 9 × 7.5 cm mass medial to the left kidney. A follow-up computed tomography (CT) scan showed an enhancing 9 × 6 cm mass anterior and medial to the left kidney. Laboratory studies revealed a normal dexamethasone suppression test and an elevated androstenedione level of 182 ng/dl. Given these results, an androgen-producing adrenal tumor was suspected. The differential diagnosis also included pheochromocytoma, lymphoma, and mesenteric gastrointestinal stromal tumor. The patient underwent robotic-assisted left adrenalectomy. The intraoperative finding of “focal invasion” into the renal parenchyma raised the possibility of adrenal cortical carcinoma; therefore, an additional left upper pole partial nephrectomy was performed. The specimen received was an 11 × 7.2 × 6.8 cm adrenal mass with attached portion of kidney. The mass was golden yellow, well circumscribed, and grossly adherent to the kidney. Histologic evaluation revealed an adrenal cortical adenoma without any features of malignancy, with a Weiss score of 0 (Fig. 1). The adjacent adrenal parenchyma shared an incomplete capsule with the kidney and was in direct contact with the renal cortex, establishing the diagnosis of fusion between the two organs (Fig. 2). The postoperative course was uneventful.

## Discussion

The true incidence of this anomaly is unknown. To the best of our knowledge, just 18 cases have been reported in the literature to date. Those patients and our present case are summarized in Table 1. The age of the patients ranged from 41 to 83 years old, including eight males and eleven females. Ten cases occurred on the right side, seven cases occurred on the left side, and two cases involved bilateral kidneys and adrenal glands. Due to the normal anatomical location of the adrenal gland, renal–adrenal fusion tends to occur at the upper pole of the kidney.

**Table 1:** Summary of renal–adrenal fusion reported in the literature

Reference	Age/sex	Procedure	Initial clinical impression	Laterality	Final diagnosis in addition to renal–adrenal fusion
Honore *et al*. [2]	47/F	NA (postmortem examination)	NA	R	NA
Ye *et al*. [3]	51/M	Radical nephrectomy	Renal cell carcinoma	L	Papillary renal cell carcinoma
Ye *et al*. [3]	49/F	Partial nephrectomy	Adrenal cortical adenoma	L	Adrenal cortical adenoma
Colberg *et al*. [4]	83/F	Partial nephrectomy with adrenalectomy	Renal mass	R	Adrenal heterotopia
Fan *et al*. [5]	62/M	Radical nephrectomy	Renal cyst	R	Adrenal Pseudocyst
Mahadevia *et al*. [6]	76/F	Partial nephrectomy with adrenalectomy	Renal mass	L	Adrenal cortical adenoma
James *et al*. [7]	54/M	Adrenalectomy	Primary hyperaldosteronism	R	Adrenal cortical adenoma
James *et al*. [7]	41/F	NA	Renal mass	R	Adrenal cortical neoplasm
James *et al*. [7]	45/F	NA	Renal mass	R	Clear cell renal cell carcinoma
James *et al*. [7]	68/M	NA	Renal mass	R	Clear cell renal cell carcinoma and renal hematoma
James *et al*. [7]	43/F	NA	Renal cortical cyst	R	Cortical cyst
St Clair *et al*. [8]	61/M	Partial nephrectomy with adrenalectomy	Renal mass	L	Adrenal cortical adenoma
Patel *et al*. [9]	70/F	Partial nephrectomy	Renal mass	L	Intrarenal adrenal cortical adenoma
Miller *et al*. [10]	62/F	Partial nephrectomy with adrenalectomy	Cystic renal mass	L	Adrenal cortical adenoma and ectopic adrenal tissue
Bamford *et al*. [11]	55/M	NA (radiological diagnosis)	Urothelial carcinoma	Bilateral	NA
Basourakos *et al*. [12]	61/M	Radical nephrectomy	Renal mass	R	Pheochromocytoma
Schwenke *et al*. [13]	48/F	NA (radiological diagnosis)	Renal mass	Bilateral	Right adrenal myelolipoma
Boll *et al*. [14]	59/M	Adrenalectomy with partial nephrectomy	Adrenal cortical adenoma	R	Adrenal cortical adenoma
Present case	77/F	Adrenalectomy with partial nephrectomy	Adrenal mass	L	Adrenal cortical adenoma

*NA* not applicable/not available

Renal–adrenal fusion is typically an incidental finding, since there are no clinical symptoms that are associated with this condition. A majority of reported cases were identified after surgical resection for adrenal or renal neoplasms. However, it is important to know that this rare anomaly can cause confusion on CT and magnetic resonance imaging scans, particularly when there is a concurrent adrenal adenoma, which may appear as an infiltrative mass on radiology, leading to misdiagnosis and unnecessary surgical procedures, for example, partial or even radical nephrectomy [5, 6]. Bamford *et al*. described that the characteristic findings on a CT scan include lack of a discrete fat plane between the upper pole of the kidney and adrenal gland, with or without a contiguous well-defined lesion within the adjacent kidney. These findings are not specific, and it is difficult to exclude an invasive renal, adrenal, or retroperitoneal lesion in unilateral cases [11]. Two of the radiologically diagnosed cases were both bilateral, and as reported by Bamford *et al*., the striking symmetry of the appearances and lack of suspicious uptake on positron emission tomography (PET)–CT helped to render the diagnosis [11, 13].

Renal–adrenal fusion can also pose a challenge from a surgical perspective. Boll *et al*. described a case of renal–adrenal fusion identified during a laparoscopic right adrenalectomy for adenoma [14]. They found that the normally avascular plane between the adrenal and renal capsule was absent, and instead, there were dense fibrotic adhesions. Those intraoperative findings may be concerning for invasive malignancy, which would require a more extensive resection. Moreover, the intraoperative frozen section of the fused adrenal tissue may be misinterpreted as renal cell carcinoma resulting in a radical nephrectomy, as reported by Fan *et al*. [5].

The histological diagnosis of a renal–adrenal fusion on permanent resection specimens is relatively straightforward. Findings of adherence of normal adrenal and renal parenchyma without a complete capsule allow for a confident diagnosis.

## Conclusion

Renal–adrenal fusion is a rare entity. The adherence of those two organs can pose a diagnostic challenge on imaging studies and also cause confusion intraoperatively. It is important to be aware of this uncommon anomaly to avoid misdiagnosis and unnecessary resection.

## Data Availability

Not applicable.

## Abbreviations

**CT**: Computed tomography
**PET**: Positron emission tomography
